# CONVERGENT CELL NONAUTONOMOUS PATHWAYS REWIRE METABOLISM TO SLOW AGING

**DOI:** 10.1093/geroni/igac059.1733

**Published:** 2022-12-20

**Authors:** Scott Leiser, Shijiao Huang, Hillary Miller, Safa Beydoun, Elizabeth Dean, Hyo Choi, Ajay Bhat, Marshall Howington

**Affiliations:** University of Michigan, Ann Arbor, Michigan, United States; University of Michigan, Ann Arbor, Michigan, United States; University of Michigan, Ann Arbor, Michigan, United States; University of Michigan, Ann Arbor, Michigan, United States; University of Michigan, Ann Arbor, Michigan, United States; University of Michigan, Ann Arbor, Michigan, United States; University of Michigan, Ann Arbor, Michigan, United States; University of Michigan, Ann Arbor, Michigan, United States

## Abstract

An organism’s ability to perceive and respond to changes in its environment is crucial for its health and survival. Our approach to identify molecular mechanisms of aging is to focus on common mechanisms downstream of multiple pathways. This approach led to our discovery of a gene, flavin-containing monooxygenase (fmo)-2, that is both necessary and sufficient to increase lifespan and healthspan downstream of several longevity interventions, including dietary restriction and hypoxia. Surprisingly, we also find that in both hypoxia and dietary restriction models, fmo-2 is induced by cell non-autonomous signaling pathways, consistent with the worms’ perceiving the stress (e.g. low oxygen, lack of food) and changing physiology as a result. Our current work focuses on 1) the signaling networks that regulate stress perception and integrate multiple signals to change physiology, and 2) the mechanism of FMO-2-mediated longevity. Our new data suggest that these cell non autonomous networks pathways utilize both overlapping and distinct signaling mechanisms to converge on upregulation of the same gene. They also suggest that these pathways can be manipulated by small molecule drugs to increase lifespan by “tricking” the organism into activating stress response networks. We further find that FMO enzyme expression has a drastic effect on endogenous metabolism, primarily through tryptophan and one carbon metabolism. Ultimately, we aim to leverage our results in a translational framework to identify key signals, genes, and mechanisms where organisms respond to the perception of environmental stress to improve health and slow aging.

